# The Impact of Regular Self-weighing on Weight Management: A Systematic Literature Review

**DOI:** 10.1186/1479-5868-5-54

**Published:** 2008-11-04

**Authors:** Jeffrey J VanWormer, Simone A French, Mark A Pereira, Ericka M Welsh

**Affiliations:** 1 Department of Education, Minneapolis Heart Institute Foundation, 920 East 28th St, Suite 100, Minneapolis, MN 55407, USA; 2 Division of Epidemiology & Community Health, University of Minnesota, Room 300 WBOB, 1300 South 2nd Street, Minneapolis, MN 55454, USA

## Abstract

**Background:**

Regular self-weighing has been a focus of attention recently in the obesity literature. It has received conflicting endorsement in that some researchers and practitioners recommend it as a key behavioral strategy for weight management, while others caution against its use due to its potential to cause negative psychological consequences associated with weight management failure. The evidence on frequent self-weighing, however, has not yet been synthesized. The purpose of this paper is to evaluate the evidence regarding the use of regular self-weighing for both weight loss and weight maintenance.

**Methods:**

A systematic literature review was conducted using the MEDLINE, CINAHL, and PsycINFO online databases. Reviewed studies were broken down by sample characteristics, predictors/conditions, dependent measures, findings, and evidence grade.

**Results:**

Twelve studies met the inclusion/exclusion criteria, but nearly half received low evidence grades in terms of methodological quality. Findings from 11 of the 12 reviewed studies indicated that more frequent self-weighing was associated with greater weight loss or weight gain prevention. Specifically, individuals who reported self-weighing weekly or daily, typically over a period of several months, held a 1 to 3 kg/m^2 ^(current) advantage over individuals who did not self-weigh frequently. The effects of self-weighing in experimental studies, especially those where self-weighing behaviors could be isolated, were less clear.

**Conclusion:**

Based on the consistency of the evidence reviewed, frequent self-weighing, at the very least, seems to be a good predictor of moderate weight loss, less weight regain, or the avoidance of initial weight gain in adults. More targeted research is needed in this area to determine the causal role of frequent self-weighing in weight loss/weight gain prevention programs. Other open questions to be pursued include the optimal dose of self-weighing, as well as the risks posed for negative psychological consequences.

## Background

Excess body weight is a leading cause of death in the U.S. [1,2], contributing to the development or complication of many chronic diseases including heart disease, diabetes, and cancer [3,4] Fortunately, even a modest amount of weight loss has been shown to reduce the incidence of chronic diseases and improve obesity-related health conditions [5]. Many people who have experienced weight issues have learned to manage their weight over the long-term via sustained, moderate caloric restriction and regular physical activity [6]. Poor adherence to these behaviors, however, is the norm for individuals attempting to lose weight or maintain weight loss [7] due to substantial physiological [8], environmental [9-11], and motivational barriers [12].

Because of the inherent challenges associated with losing weight or preventing weight regain, many people turn to some form of external assistance (e.g., clinical counseling program, community support group, self-help book) to help them initiate or maintain the behavior changes required to lose weight. A major component of these forms of assistance involves instruction in behavioral self-management skills like goal-setting or stimulus control. In particular, regular self-monitoring of weight has been recommended as a key component of behavioral self-regulation of body weight [5].

Regular self-weighing seems to be a common strategy for individuals who have been successful at losing weight and keeping it off. Klem and colleagues [13] found that 75 percent of a cohort of weight loss maintainers report self-weighing at least weekly. Weekly self-weighing also seems to be more common among individuals who lost weight on their own versus using an organized weight management program [14]. In contrast, the prevalence of self-weighing in the general population of healthy weight individuals is not well studied, but one study estimated that about 39 percent self-weigh weekly [15].

Frequent self-weighing is conceptualized to work via behavioral self-regulation [16]. Specifically, an individual who self-weighs often is believed to stay focused on and sensitive to changes in their weight. This creates more opportunities for self-reinforcement of even small weight loss (or weight maintenance) progress. Also, the individual is empowered to quickly identify lapses in their progress and adjust their behavior accordingly to head off substantial weight gain [17].

Despite a plausible rationale, there remains considerable debate on the utility of self-weighing in the context of weight management. Some researchers and practitioners urge caution in the use of frequent self-weighing, at least with some individuals, because it is believed to produce negative psychological conditions such as depression, anxiety, or otherwise unhealthy preoccupations and stress associated with weight [18,19]. Furthermore, the downstream effect of these psychological conditions produced by frequent self-weighing is believed to undermine the effectiveness of weight management interventions by negatively influencing body image and increasing program attrition. Others, however, have noted that most investigations that have observed negative psychological harms secondary to frequent self-weighing have done so only in non-overweight samples [20] and several studies have shown a strong positive association between self-weighing frequency and magnitude of weight loss.

The findings on the utility of regular self-weighing for weight management have yet to be critically reviewed or synthesized. Therefore, the purpose of this paper was to conduct a systematic review of the literature from observational and experimental studies on self-weighing in order to gauge the effectiveness of regular self-weighing on weight loss and weight maintenance (including primary weight gain prevention) in adults. The central research questions examined are: (1) Do the benefits of frequent self-weighing, in terms of body weight, outweigh the disadvantages, and (2) Do the conclusions in this regard differ by the subgroups of individuals who are interested in weight loss or weight maintenance? We hypothesize that the preponderance of evidence would support the use of frequent self-weighing behaviors to promote both weight loss and prevention of weight (re)gain. Implications in the context of weight loss research and clinical practice are also discussed.

## Methods

A systematic review of the literature was conducted. MEDLINE, CINAHL, and PsycINFO online databases were searched via the University of Minnesota's Ovid interface  to produce relevant articles on self-weighing and weight management. The reference sections of all included studies were also manually searched.

### Inclusion and Exclusion Criteria

Inclusion criteria were: English language, adult participants, assessment of body weight, assessment of self-weighing frequency (i.e., used as a treatment component, predictor, or outcome variable), quantitative analysis of the relationship between self-weighing and body weight, and published before January 1, 2008. Since the research question was relatively broad and the body of evidence was expected to be small, no restrictions were placed on sample size, setting, research design, body weight of participants, or length of measurement follow-up. The MEDLINE search included one Medical Subject Heading, Body Weight, along with two text words; self-weighing or self-monitoring. Limiters included English language, humans, and adult population. Keywords used in the PsycINFO search included obesity, body weight, weight loss, self-weighing, and self-monitoring (limiters included English language, humans, and adult population).

### Data Extraction

The outcome of interest was body weight or change in body weight. For each study, the most conservative approach to data extraction was taken by reporting only findings from the final follow-up visit, and, where possible, only those that were statistically adjusted for potential disturbance variables. To maintain the focus on the scope of the research question of interest, psychological outcomes believed to be related to self-weighing (e.g., depression, obsessive-compulsive disorder, binge eating, body image disorder) were not described. Several investigations on such negative psychological consequences secondary to self-weighing have appeared recently in the scientific literature, therefore the authors agreed this question would be best served by a separate review paper focused on that topic.

### Data Synthesis

Studies were broken down by sample characteristics, predictors/conditions, dependent measures, findings, and evidence grade. A previously used adaptation of the American Diabetes Association's (ADA) evidence grading system was used (see Table 1) [21,22]. Studies were assigned an evidence grade of A, B, or C along with a strength grade of 1, 2, or 3 depending on methodological quality, supporting evidence, and estimated benefits to the population at-risk.

**Table 1:** ADA-adapted system for grading reviewed studies.

**EVIDENCE GRADE**	**DEFINITION**	**STRENGTH RECOMMENDATION**	**DEFINITION**
A	1. Clear evidence from well-conducted, generalizeable, randomized-controlled trials that are adequately powered, including:	1	Substantial benefit to persons at-risk
	a. evidence from a multi-center trial		
	b. evidence from a meta-analysis		
	2. Supportive evidence from well-conducted, randomized-controlled trials that are adequately powered, including:		
	a. evidence from a trial at one or more institutions		
	b. evidence from a meta-analysis		

B	3. Supportive evidence from well-conducted cohort studies, including:	2	Moderate benefit to persons at-risk
	a. evidence from a prospective cohort study		
	b. evidence from a prospective registry		
	c. evidence from a meta-analysis of cohort studies		
	4. Supportive evidence from well-conducted case-control studies		

C	5. Supportive evidence from poorly controlled or uncontrolled studies, including:	3	Uncertain benefit to persons at-risk
	a. evidence from randomized clinical trials with one or more major or three or more minor methodological flaws that could confound results		
	b. evidence from observational studies with high potential for bias		
	c. evidence from case series or case reports		
	6. Conflicting results with the weight of the evidence supporting the recommendation		
	7. Expert consensus or clinical experience without support from research studies		

## Results

### Study Characteristics

**Figure 1:**
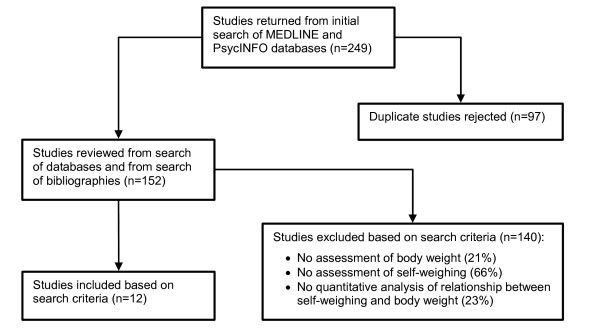
Flow diagram of the study identification, selection, and exclusion process.

As outlined in Figure 1, 249 articles were returned from the initial searches of the online databases. Twelve studies [23-34] were included in the review per the inclusion/exclusion criteria and the reasons for exclusion are also given in Figure 1. With the exception of one study that exclusively recruited males [31], all other study samples primarily included middle-aged females. Four studies recruited females exclusively [24,25,27,33] and two studies were conducted in Japan [33,34]. Median baseline body mass index (BMI) was about 30 kg/m^2 ^across all samples (see Table 2).

**Table 2:** Synopsis of reviewed studies on self-weighing and weight management.

**REFERENCE, SAMPLE, DESIGN, & FOCUS**	**PREDICTORS OR CONDITIONS**	**OUTCOME MEASURES**	**FINDINGS**	**EVIDENCE GRADE & COMMENTS**
Butryn, et al. (2007) ^23^ 3,003 participants enrolled in the U.S. National Weight Control Registry for at least one year. The mean age was 48 years and 75% of the sample was female. Mean baseline BMI was 25 kg/m^2^. Prospective cohort Weight maintenance	To be included as part of the National Weight Control Registry, participants had to have maintained at least a 30 pound weight loss over one year. Predictors included: 1) Self-weighing frequency (At least daily, At least weekly, Less than weekly) 2) Change in self-weighing frequency (Increase, No change, Decrease)	Outcomes were assessed at baseline and 12-months follow-up. 1) ΔBody weight (kg)	2,462 (82%) participants had complete data from both the baseline and 12-month assessments. Compared to participants who increased (1.2) or did not change their self-weighing frequency (1.7), participants who decreased (3.7) their self-weighing frequency had significantly greater weight gain at the 12-month follow-up.	B2 – After adjustment for several potential confounders, there was benefit for weight maintenance by increasing the frequency of self-weighing over 12 months. Potential biases, however, may have been introduced by the self-reported weight measures and exclusion of 18% of the full sample due to missing data.

Qi, et al. (2000) ^24^ 50 obese, postmenopausal female participants recruited in Maryland. The mean age was 60 years. Mean baseline BMI was approximately 32 kg/m^2^. Prospective cohort Weight loss	Participants completed a 6-month behavioral weight loss intervention. After the weight loss program, participants were then stratified by the predictors: 1) Weight loss (> 5 kg, ≤ 5 kg)	Outcomes were assessed at baseline and 6-months follow-up. They were reported as pre-post-scores (versus change scores) 1) Daily self-weighing score (1-less frequent to 5-more frequent)	50 (100%) participants were available for the 6-month follow-up. Participants who lost > 5 kg during treatment observed a significant pre-post increase in their daily self-weighing score (1.7 to 2.5).	C3 – Participants who lost > 5 kg had significantly increased their frequency of self-weighing, but post scores were statistically indistinguishable between groups. Interpretation of the findings was complicated because only the average scaled scores for self-weighing were reported (versus response distributions). Also, no multivariate adjustments were made in the analysis despite the small sample and several significant baseline differences between groups.

McGuire, et al. (2007) ^25^ 500 participants recruited through a random-digit phone survey in the U.S. The mean age was 46 years and 59% of the sample was female. Mean BMI was between 25 and 30 kg/m^2^. Cross-sectional Weight maintenance	1) Weight loss maintainer – lost ≥ 10% of maximum weight and currently at this level for ≥ 1 year 2) Weight loss regainers – lost ≥ 10% of maximum weight, but not currently at this level 3) Controls – never lost ≥ 10% of maximum weight and never weighed ≥ 10% of current weight	Outcomes were assessed at survey completion. 1) At least weekly self-weighing (%)	The overall survey response rate was 57% and 238 participants had complete data for the analysis. Compared to Controls (34.5) and Weight loss regainers (35.7), a significantly greater proportion of Weight loss maintainers (55.1) self-weighed at least weekly.	C2 – After adjustment for several potential confounders, there were significantly more weight loss maintainers that reported weekly self-weighing. The cross-sectional nature of the study limited conclusions on cause-and-effect. Also, potential biases may have been present with the self-reported operational definition and measurement of weight maintenance, much of it being rather complex to recall.

Linde, et al. (2005) ^26^ Two separate samples were analyzed. Sample 1 consisted of 1,226 participants enrolled in the Pound of Prevention trial in Minnesota. The mean age was 35 years and 81% of the sample was female. Baseline BMI was 27 kg/m^2^. Sample 2 consisted of 1,800 participants enrolled in the Weigh to Be trial in Minnesota. The mean age was 51 years and 72% of the sample was female. Mean baseline BMI was 34 kg/m^2^. Prospective cohort Weight loss and weight maintenance	The Pound of Prevention trial involved a general population with an intervention focused on weight gain prevention. The Weigh to Be trial involved an overweight population with a telephone-based intervention focused on weight loss. Predictors included: 1) Self-weighing frequency (Never, Semi-monthly, Monthly, Weekly, Daily)	Outcomes were assessed at baseline, 12-, and 24-months follow-up for both samples. 1) ΔBMI (kg/m^2^)	In the Pound of Prevention sample, 992 (81%) participants were available for the 24-month follow-up. Participants who self-weighed daily (-0.8) lost significantly more body mass relative to participants who self-weighed weekly (0.3), monthly (0.8), semi-monthly (0.8), or never (1.1). Also, participants who self-weighed weekly gained significantly less body mass relative to participants who self-weighed monthly, semi-monthly, or never. In the Weigh to Be sample, 1,000 (56%) participants were available for the 24-month follow-up. Participants who self-weighed daily (-1.9) lost significantly more body mass relative to participants who self-weighed weekly (-1.0), monthly (-0.2), semi-monthly (0.2), or never (0.8). Also, participants who self-weighed weekly lost significantly more body mass relative to participants who self-weighed monthly, semi-monthly, or never. Participants who never self-weighed gained significantly more weight relative to participants who self-weighed semi-monthly or monthly. *Note that findings were estimated from study graphs because precise means were not reported*.	B1 – After adjustment for several potential confounders, there was clear benefit for both weight maintenance and weight loss with more frequent self-weighing reported at the 24-month follow-ups. The long follow-up and large sample sizes were strengths in both samples, but attrition bias was especially concerning in the Weight to Be sample.

Linde, et al. (2007) ^27^ 4,660 female participants recruited from a health plan in Washington. The mean age was 52 years and the mean BMI was 28 kg/m^2^. Cross-sectional Weight in general	1) Self-weighing frequency (Never, Monthly, Weekly, Daily)	Outcomes were assessed at survey completion. 1) BMI (kg/m^2^)	The overall survey response rate was 62% and 4,581 participants had complete data for the analysis. Compared to participants who self-weighed daily (29.2), participants who self-weighed weekly (30.1), monthly (30.6), and never (30.9) had significantly higher BMI's. Also, participants who self-weighed weekly had significantly lower BMI's relative to participants who never self-weighed.	C2 – After adjustment for several potential confounders, more frequent self-weighing was associated with significantly, though modestly, lower BMI. The cross-sectional nature of the study and the reliance on self-report measures limits validity and any conclusions on cause-and-effect. Also, the low response rate may hamper generalizability.

Levitsky, et al. (2006) ^28^ Two separate experiments were conducted. In experiment 1, the sample consisted of 32 female freshman students recruited from introductory college classes in New York. Age ranged between 18 and 21. Baseline BMI was not reported, but weight was 63 kg. In experiment 2, the sample consisted of 41 female freshman college students recruited via school advertisements and classroom announcements in New York. Age was greater than 18 years. Baseline BMI was not reported, but weight was 62 kg. Randomized-controlled trials Weight maintenance	In experiment 1: 1) Experimental - • Basic nutrition information • Home scale provided, as well as instruction to weigh daily and e-mail observed weight to study staff • Daily e-mail feedback on body mass change 2) Control - • Basic nutrition information In experiment 2: 1) Experimental - • Basic nutrition information • Home scale provided, as well as instruction to weigh daily and e-mail observed weight to study staff • Daily e-mail feedback on recommended calorie consumption to maintain weight 2) Control - • Assessment only	Outcomes were assessed at enrollment (ie, first week of class) and post-semester (ie, last week of class; ~10 weeks) for both samples. 1) ΔBody weight (kg)	In experiment 1, 26 (81%) participants were available for the post-semester follow-up. Participants in the Experimental group (0.1) gained significantly less weight relative to Controls (3.1). In experiment 2, 32 (78%) participants were available for the post-semester follow-up. Participants in the Control group (2.0) gained significantly more weight relative to the Experimental group (-0.8).	B1 – Groups that employed a frequent self-weighing treatment gained less weight relative to groups who received minimal contact. These results were essentially replicated in both experiments. The experimental designs were limited, however, by the samples, which were small, quite homogenous, and not particularly well described.

Kruger, et al. (2006) ^29^ 4,345 respondents from the HealthStyles survey, recruited through a representative consumer database in the U.S. In the analytical sample, the median age was between 45 and 64 years, and 62% were female. Median BMI was between 30 and 35 kg/m^2^. Cross-sectional Weight maintenance	1) Weight loser – reported lost weight and kept it off (Successful, Non-successful)	Outcomes were assessed at survey completion. 1) Daily self-weighing (%)	The overall survey response rate was 70% and 1,958 participants had complete data for the analysis and fit into the predictor categories. A significantly greater proportion of Successful weight losers (20) reported self-weighing daily relative to non-successful weight losers (11).	C3 – After adjustment for potential confounders, there were significantly more successful weight losers that reported daily self-weighing. There were several potential biases, however, in regard to the vague measurement and definition of a successful weight loser, as well as the exclusion of many respondents who were not believed to fit this definition.

Wing, et al. (2006) ^30^ 314 participants who lost at least 10% of their body weight over the previous two years. The sample was recruited through newspaper advertisements, brochures, and weight loss program contacts in Rhode Island. The mean age was 51 years and 81% of the sample was female. Mean baseline BMI was approximately 29 kg/m^2^. Randomized-controlled trial Weight maintenance	Study conditions included: 1) Internet - • Tool kit with self-monitoring diaries, pedometer, and several cans of Slim-Fast meal replacements • Home scale, laptop computer, and Internet connection and instructed to submit weight and physical activity monitoring data to the study website weekly • Immediate Internet feedback based on submitted weight • Monthly (weekly during the first month) web-based treatment lessons led by Master's or PhD level nutritionists, exercise physiologists, or clinical psychologists • Offered additional individual e-counseling if desired (to return to goal weight) • Access to message board on the website 2) Face-to-face - • Same as the Internet group, but weight information was sent over an automated telephone system and weekly counselling sessions were face-to-face 3) Control - • Quarterly newsletter with information on diet, exercise, and weight loss Secondary analytical predictors: 1) Self-weighing frequency (Daily, Less than daily)	Outcomes were assessed at baseline, 6-, 12-, and 18-months follow-up. 1) ≥ 2.3 kg weight regain (%)	291 (93%) participants completed the 18-month follow-up. Secondary analyses revealed that, within the Internet group, a significantly smaller proportion of participants who self-weighed daily (40) regained ≥ 2.3 kg relative to participants who did not self-weigh daily (68). Within the Face-to-face group, a significantly smaller proportion of participants who self-weighed daily (26) regained ≥ 2.3 kg relative to participants who did not self-weigh daily (58).	A2 – Both treatment groups, which involved frequent self-weighing, decreased the proportion of participants who regained at least 2.3 kg, but only the Face-to-face group significantly reduced the amount of total weight regained after 18 months. Within both treatment groups, daily self-weighing in particular predicted a significantly smaller proportion of participants who regained at least 2.3 kg. This study had several strengths including random assignment, a large sample size, multiple comparison groups, and very clear measures. It also suggested that the benefits of self-weighing may depend on the accompanying level of programmatic support, but the effects of self-weighing could not be isolated given all the other treatment components.

Jeffery, et al. (1984) ^31^ 89 obese male participants recruited in Minnesota. The mean age was 53 years. Mean baseline BMI was approximately 32 kg/m^2^. Prospective cohort Weight loss	Participants completed a 15-week, group-counseling based behavioral weight loss intervention. Predictors included: 1) Self-weighing frequency (Daily, Less than daily)	Outcomes were assessed at baseline, post-treatment, 1-year, and 2-years. 1) ΔBody weight (lb)	81 (91%) participants were available for the 2-year follow-up. Participants who self-weighed daily (-17.1) lost significantly more weight at the 2-year follow-up relative to participants who self-weighed less than daily (-6.7).	B2 – There was significant weight loss benefits to participants who self-weighed daily at 2-years follow-up. Strengths included the long follow-up period. The analysis of self-weighing was done in a univariate fashion, however, and it was not clear if self-weighing was beneficial beyond the 1-year follow-up in the multivariate analyses that accounted for confounders.

Heckerman, et al. (1978) ^32^ 23 overweight participants recruited in Rhode Island. The mean age was 47 years and 87% of the sample was female. Mean baseline BMI was not reported, but participants averaged 72% overweight. Randomized-controlled trial Weight loss	Study conditions included: 1) Weigh-in - • Weekly weigh-ins for 10 weeks, followed by monthly weigh-ins for 6 months • Weekly group-based treatment sessions for 10 weeks followed by monthly treatment sessions for 6 months • Treatment sessions included instruction in stimulus control, self-monitoring, and eating/exercise advice • Instruction to self-weigh frequently between treatment sessions 2) No Weigh-in - • Same as the Weigh-in group, but weigh-ins only conducted at baseline, as well as the end of the treatment and follow-up phases • Instructed not to self-weigh between sessions	Outcomes were assessed at baseline, 10-, and 34-weeks follow-up. 1) ΔBody weight (lb)	7 (30%) participants completed the 34-week follow-up. No significant differences were observed.	B3 – Participants in the No Weigh-in group actually lost more weight and were more likely to attend the follow-up visits, but the study was severely limited by a small sample size and attrition bias.

Tanaka, et al. (2004) ^33^ 262 overweight female participants recruited from a hospital weight loss program in Japan. From the analytical sample, the mean age was 49 years. Mean baseline BMI was 29 kg/m^2^. Prospective cohort Weight loss	Participants were offered a 16-week nutrition education program that recommended a 1400 kcal/d diet. During this program, participants were advised to self-graph weight 4 times daily on a week-long graph. Predictors included: 1) Time (0-, 16-weeks follow-up)	Outcomes were assessed at 0-, 4-, 8-, 12-, and 16-weeks follow-up. 1) Body weight (log kg)	98 (37%) participants had complete data for analysis (ie, completed adequate self-weighing and completed weight control program). Body weight at 16-weeks (4.17) was significantly lower than 0-weeks (4.24).	C3 – Participants who received the intervention, which included a very frequent self-weighing component, lost significant weight over 16 weeks. Potential biases, however, were likely as a result of high attrition and the exclusion from the analysis of participants who could have served as controls (ie, those who did not self-weigh enough or failed to complete the weight loss program). Also, participants served as their own controls.

Fujimoto, et al. (1992) ^34^ 89 obese participants, recruited through a hospital treatment program in Japan. The mean age was approximately 43 years and 83% of the sample was female. Mean baseline BMI ranged between 31 and 34 kg/m^2^. The analytical sample was stratified by sex and those that completed two years of follow-up. Randomized-controlled trial Weight loss	Study conditions included: 1) Behavior Therapy plus Charting - • Recommendation to self-graph weight 4 times daily on a week-long graph • Complete regular food diary • Weekly or biweekly interviews (~6 month duration) with hospital physician to review weight graphs, food diaries, and discuss food and fluid intake. 2) Behavior Therapy Alone - • Same as above, but without the daily graphing component	Outcomes were assessed at enrollment, post-treatment, and 2-years follow-up. 1) ΔBody weight (kg)	59 (66%) of the 74 female participants were available for the 2-year follow-up. It was unclear how many males were available for the 2-year follow-up. In the female sub-sample, the Behavior Therapy plus Charting group (-14.9) lost significantly more weight relative to the Behavior Therapy Alone group (-7.8) after two years.	B2 – The intervention was beneficial over 2 years in that the group that included weight charting 4 times daily lost significantly more weight relative to the group that received behavior therapy alone. Strengths included the long follow-up period. Generalizability may be questionable, however, given the intensity of the self-weighing protocol and the lack of process data documenting the observed (versus assigned) frequency of self-weighing. Also, methodological weaknesses included the vague description of some treatment procedures and stratification of the sample that severely reduced power.

The reviewed studies were almost evenly split in their focus on either weight loss or weight maintenance. Body weight was self-reported in all three cross-sectional studies and one of the prospective cohort studies, while all other studies used objective assessments of body weight. Self-weighing, when assessed independently as a predictor variable (versus implicitly as part of a treatment package), was done so exclusively by self-report.

### Research Quality

Using the ADA-adapted evidence grading system [21,22], only one study provided A-level evidence in terms of methodological quality [30]. All other studies provided B- or C-level evidence, primarily due to weaker research designs, high non-response or loss to follow-up, underpowered samples, and/or incomplete statistical analyses. With strength grades of 1, the studies by Linde et al. [26] and Levitsky et al. [28] provided the strongest (positive) associations between self-weighing and weight management. Six studies received strength grades of 2, while the remaining four studies received strength grades of 3.

### Self-weighing and Weight Maintenance

All three cross-sectional studies indicated a significant association between the frequency of self-weighing and body weight. Specifically, nationally representative samples revealed that, relative to respondents who did not maintain their weight loss, about 60 [25] and 80 [29] percent more respondents who were successful at keeping their weight off reported weekly or daily self-weighing, respectively. The study by Linde et al. [27] indicated that women who reported daily self-weighing weighed nearly 2 BMI units less than women who reported never self-weighing.

Similar to the results observed in her cross-sectional analysis [27], Linde et al. [26] also found that participants in the Pound of Prevention [15] cohort analysis who reported daily self-weighing at the two-year follow-up weighed nearly 2 BMI units less than participants who reported never self-weighing. Butryn et al. [23] found that participants who increased their frequency of self-weighing (unspecified magnitude of increase) over one year gained 2.5 kg less weight relative to participants who decreased their frequency of self-weighing over this same time period.

Two randomized-controlled trials focused on weight maintenance. Secondary analyses by Wing and colleagues [30] found that, in both study treatment conditions, 41 to 55 percent fewer participants who self-weighed daily regained ≥ 2.3 kg relative to participants who did not self-weigh daily. In two separate, short-term experiments conducted by Levitsky et al. [28] a 3 kg weight advantage was noted for college females who received a daily self-weighing and feedback intervention relative to participants who received information-only or assessment-only treatments.

### Self-weighing and Weight Loss

In terms of weight loss, a prospective cohort analyses by Linde et al. [26] found that participants in the Weigh To Be trial [35] who self-weighed daily lost about 1 BMI unit more than participants who self-weighed weekly and nearly 3 BMI units more than participants who did not self-weigh at all. Jeffery and colleagues [31] found that, after two years, participants in a 15-week behavioral weight loss program who reported daily self-weighing lost about 15 pounds more than participants who reported self-weighing less than daily. Qi et al. [24] found that, compared to participants who did not lost 5 kg or more, participants who lost 5 kg or more in a behavioral weight loss program significantly increased their daily self-weighing frequency between baseline and 6-months follow-up. An uncontrolled cohort study by Tanaka and colleagues [33] found that participants who completed a nutrition-focused weight loss program whereby they self-weighed 4 times per day lost a significant amount of weight (~4 kg) over 16 weeks.

Two randomized-controlled trials isolated the effects of frequent self-weighing on weight loss by comparing it to an identical intervention that contained everything but the self-weighing component. These two trials found conflicting results. Heckerman and colleagues [32] found no advantage of frequent self-weighing above and beyond a standard 10-week behavioral weight loss program. Fujimoto and colleagues [34], however, found that a group that self-weighed very frequently (4 times daily) lost twice as much weight over a two year period relative to a group that received behavioral weight loss therapy alone.

## Discussion

In all but one of the twelve reviewed studies, frequent self-weighing (defined as self-weighing weekly or daily), or treatment groups that utilized frequent self-weighing, was associated with significantly greater weight loss, weight maintenance, or less body weight in general relative to infrequent self-weighing. Generally speaking, weekly and daily self-weighers held approximately a 1 and 2 BMI unit advantage, respectively, over never self-weighers [26,27]. In regard to weight loss, weekly and daily self-weighers lost about 2 to 3 BMI units (~12-18 pounds) more than participants who did not weigh as frequently [26,31]. Based largely on the consistency of the evidence reviewed, frequent self-weighing, at the very least, seems to be a good predictor of moderate weight loss and weight maintenance, both for individuals who have lost weight and are attempting to keep it off and for individuals who are attempting to avoid weight gain in the first place.

Only three studies [28,32,34] directly tested a self-weighing intervention that was not part of an extensive treatment package, relative to control groups that did not receive self-weighing advice or support. These trials were small, showed conflicting results, and raised concerns over internal and external validity. Levitsky and colleagues [28] utilized a brief self-weighing intervention for weight maintenance among female freshman college students (generally non-overweight) and found about 1 BMI unit less weight gain for intervention participants relative to controls. Heckerman, et al. [32] found no significant weight loss advantage for participants enrolled in a 10-week behavioral weight loss program that included weekly weigh-ins relative to a group that received the same treatment program without weekly weigh-ins. Attrition was extremely high in this small sample, however, and few conclusions could be drawn from the results. In a somewhat similar approach, Fujimoto et al. [34] also tested a behavioral weight loss program with and without frequent self-weighing. The self-weighing in this study included a recommendation to chart one's weight four times per day. The findings indicated a strong effect in that the self-weighing group lost nearly twice as much weight as compared to the group that did not self-weigh. Analyses from this study, however, were difficult to interpret due to the exclusion of a large part of the randomized sample. As such, selection bias may have been present.

An ideal objective of this review would be to draw conclusions on the optimal dose of self-weighing. At this time, however, the evidence base does not support endorsement of a precise self-weighing frequency and duration that has the most benefit for the most people. In terms of a threshold, weekly self-weighing over several months stands out as what may be the minimum point at which meaningful weight benefits begin to accrue. This assertion is primarily based on the Linde et al. studies [26,27], which were the only ones with enough power to retain a precise assessment of self-weighing frequency across several levels (e.g., daily, weekly, monthly, rarely) versus a dichotomized characterization (e.g., daily, less than daily). These studies found statistically significant benefit, in terms of weight loss, weight maintenance, and weight in general, beginning at weekly self-weighing. It was not clear from any of the reviewed studies if more than daily self-weighing confers added weight benefits.

Perhaps the most significant methodological limitation of the reviewed studies involved the potential for measurement bias. Self-weighing was assessed exclusively by self-report. In order to prevent recall bias, questionnaire items can not be practically designed to examine self-weighing in a timeframe that extends far beyond the point at which the question is asked. As such, the characterization of self-weighing reported may not accurately reflect self-weighing over the time periods they are deemed to represent. In other words, reported self-weighing frequency at the end of a study may not truly represent the degree to which self-weighing actually occurred over the course of the entire study (or in the months since the last follow-up visit). More objective means of assessing self-weighing frequency, such as scales that record time/date of weigh-ins at home, are needed to validate self-reported measures. Also, the overall demographic profile of study samples was somewhat narrow, primarily involving middle-aged American female volunteers. This seems to be the group most likely to present for weight management services, but it limits generalizations on the effects of self-weighing across the general population.

Although frequent self-weighing was included as part of a treatment package in one large, well-conducted, randomized-controlled trial [30], only three studies were able to experimentally isolate or disaggregate the effects of frequent self-weighing. These studies were small and contained several methodological flaws, however, and therefore strong conclusions could not be drawn. Results from the cross-sectional and prospective cohort studies are also insufficient to make causal claims due to temporality and selection bias issues. Given the controversial endorsement of frequent self-weighing in the scientific community [18,19], it seems timely to experimentally investigate different frequencies and durations of self-weighing in a diverse sample using a large randomized-controlled trial. Also, based on the differential associations of self-weighing across different treatment intensities employed by Wing and colleagues [30], the interaction between frequent self-weighing and other weight management program components would be useful for practitioners to better understand. Furthermore, more sensitive analyses are needed to identify the subgroups of people who benefit most from frequent self-weighing. For example, many of the weight maintenance studies reviewed had combined samples of individuals who had lost weight previously and were seeking to prevent weight regain alongside individuals who were normal weight and were seeking to prevent weight gain in the first place. There may be subtle differences in such subgroups that could help practitioners and program designers offer the most appropriate advice.

## Conclusion

In balancing the strengths and weaknesses of the evidence reviewed, frequent self-weighing seems to be a helpful strategy for adults who have been successful at losing weight, maintaining weight loss, or preventing weight gain. Furthermore, frequent self-weighing may serve as a useful component of standard weight loss treatment packages. At this time, weekly self-weighing seems to be a reasonable strategy to endorse for adults, but more research is needed to firmly establish the independent causal effect, as well as the optimal dose, both in terms of frequency and duration, of self-weighing. Also, more research needs to be done to determine if self-weighing is more or less effective in specific population subgroups and to identify the potential for psychological risks associated with very frequent self-weighing.

## Competing interests

The authors declare that they have no competing interests.

## Authors' contributions

JJV conceived of the review design and drafted the initial manuscript. SAF participated in the review design and helped draft manuscript revisions. MAP participated in the review design and helped draft manuscript revisions. EMW helped draft manuscript revisions. All authors read and approved the final manuscript.
